# Microbes and healthful longevity

**DOI:** 10.18632/aging.100969

**Published:** 2016-05-24

**Authors:** Susan E. Erdman

**Affiliations:** Division of Comparative Medicine Massachusetts Institute of Technology, Cambridge, MA 02139, USA

**Keywords:** aging, cancer, cachexia, FoxN1, oxytocin, lifespan

The popularity of hand sanitizer and antibiotics shows how we feel about bacteria: an enemy that's bad for our health. Emerging data, however, suggest just the opposite - that exposures to certain kinds of bacteria are beneficial for a long and healthy life, at least in part by inhibiting a wasting syndrome termed cachexia [1]. Cachexia, a condition defined as muscle wasting associated with chronic disease, arises during cancer and chronic obstructive pulmonary disease (COPD) with debilitating consequences resulting in premature death and creating a major public health burden. A growing body of research involving the host immune system reveals great potential for commensal bacteria to treat diseases and improve quality of life in animal hosts [2].

It's an intriguing possibility that microbes may be harnessed for a long, healthy, and meaningful life. Varian et al. (2016) tested this concept involving cancer-associated cachexia using a probiotic microbe *Lactobacillus reuteri* [1] isolated from human breast milk and previously shown to prevent diarrhea in humans [3]. The authors used a targeted infection in mouse models for proof-of-concept and mechanistic insight, rather than to focus on one type of bacteria *per se*.

**Figure 1:**
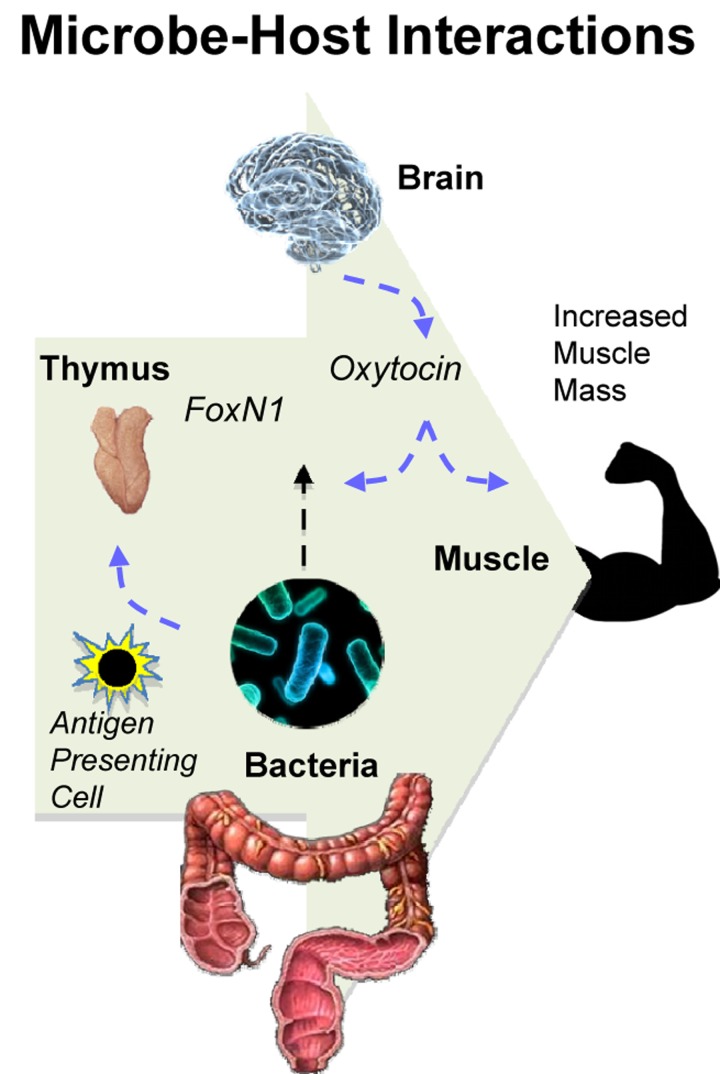
Oral supplementation with microbes in drinking water inhibits cancer-associated muscle wasting [cachexia] in mouse models. Bacteria therapy up-regulates FoxN1 and oxytocin leading to a more balanced immune system and longer lifespan.

Specifically, mice consuming probiotic *L. reuteri* were shown to have larger skeletal muscles than untreated age-matched controls (Figure 1). A surprising additional finding was increased thymus gland size only in mice consuming bacteria in their drinking water. The thymus was not only larger, but also had increased expression of Forkhead Box N1 [FoxN1], a feature involved in systemic programming of immune system lymphocytes. FoxN1 is most widely recognized as the factor missing in athymic nude mice [4]. Nude mice lack a functional thymus gland creating an immune deficit, with fewer cancer-fighting immune cells thus making them exquisitely susceptible to neoplasia. Thus, their thymic deficiency has been widely exploited in biomedical research to study cancer progression. It follows logically that mice with a larger thymus and with higher levels of FoxN1 would have increased resistance against tumors, as seen in Varian et al. 2016 [1]. Although cachexia was reduced, perhaps the most powerful finding in this study was that edible microbes globally increased FoxN1 expression even in mice without cancer, leading to a more balanced immune system during normal aging.

Bacterial stimulation of FoxN1 and increased thymus gland size has enormous implications for host good health beyond cancer and associated cachexia. Indeed, FoxN1 protein has been touted as a “Fountain of Youth” [5]. During childhood, a proficient thymus gland supplies adaptive immune cells that help fight pathogenic infections and discern self *versus* non-self, to lower risk of autoimmune diseases. With increasing age, the thymus gland naturally shrinks leading to immune dysregulation with higher risk for infections and cancer in elderly subjects. Other studies have shown that mouse models treated exogenously with FoxN1 had features of sustained youth [5]. Interestingly, animals lacking FoxN1 failed to develop larger muscles after microbial therapy [1], implicating the immune system in muscle-boosting effects.

The precise mechanism by which bacteria stimulate FoxN1 expression in thymus gland remains unknown but likely involves the *wnt* signaling pathway [1, 4]. Microbes have been shown by our own lab and by others [6] to prime the immune system for sustained good health. At the same time, the thymus gland and muscle growth may also be stimulated (delete indirectly) through bacteria-triggered upregulation of central nervous system (CNS) hormones, for example growth hormone and oxytocin [1].

The hormone oxytocin, best known for roles in childbirth and maternal nurturing [3], may be a key link between probiotic bacteria and increased muscle mass. Earlier studies showed that eating *L. reuteri* led to higher systemic levels of oxytocin with an improved wound healing capacity [3]. Oxytocin was shown separately in mice to be indispensable for healthy muscle maintenance and repair that normally declines with age [7]. One noteworthy feature of oxytocin is the ability to boost tissue regeneration without increasing risk for cancer. Not only is oxytocin proposed as an ‘Elixir of Youth’, but also as a remedy for mental health ailments including autism spectrum disorder [ASD]. Data from Poutahidis et al. (2013) links microbe-induced oxytocin with immune system function [3] in a synergistic perinatal partnership between mammalian hosts and their microbial passengers.

Perhaps exposures to bacteria can be good for us, after all. Can we formulate a bacteria cocktail that prevents cachexia and imparts a long, healthy, and meaningful life? There are certainly reasons for optimism. Human societies around the world have been consuming probiotic bacteria in fermented foods for thousands of years with few adverse health effects. Further, animal models demonstrate reproducible beneficial health outcomes throughout life using simple microbial strategies [1, 3]. Finally, tractable microbes are readily partnered with prebiotics for palatable public health benefit. Additional research is needed to explore the vast and far-reaching potential of microbes for a long and healthy life.
